# Interactional dynamics of same-sex marriage legislation in the United States

**DOI:** 10.1098/rsos.170130

**Published:** 2017-06-07

**Authors:** Subhradeep Roy, Nicole Abaid

**Affiliations:** Department of Biomedical Engineering and Mechanics (MC 0219), 495 Old Turner Street, Virginia Tech, Blacksburg, VA 24061, USA

**Keywords:** distance-based correlation, mathematical modelling, same-sex marriage, social networks

## Abstract

Understanding how people form opinions and make decisions is a complex phenomenon that depends on both personal practices and interactions. Recent availability of real-world data has enabled quantitative analysis of opinion formation, which illuminates phenomena that impact physical and social sciences. Public policies exemplify complex opinion formation spanning individual and population scales, and a timely example is the legalization of same-sex marriage in the United States. Here, we seek to understand how this issue captures the relationship between state-laws and Senate representatives subject to geographical and ideological factors. Using distance-based correlations, we study how physical proximity and state-government ideology may be used to extract patterns in state-law adoption and senatorial support of same-sex marriage. Results demonstrate that proximal states have similar opinion dynamics in both state-laws and senators’ opinions, and states with similar state-government ideology have analogous senators’ opinions. Moreover, senators’ opinions drive state-laws with a time lag. Thus, change in opinion not only results from negotiations among individuals, but also reflects inherent spatial and political similarities and temporal delays. We build a social impact model of state-law adoption in light of these results, which predicts the evolution of state-laws legalizing same-sex marriage over the last three decades.

## 1. Introduction

No issue in the United States has demonstrated the complex interaction between political and social life like same-sex marriage. 26 June 2015 was a historic day when the United States became one of 22 countries to legalize same-sex marriage across the 50 states. The decision was made by the Supreme Court, and prior to that, there were 37 states to legalize same-sex marriage independently [1]. Among these 37 states, same-sex marriage was legalized in 26 states by court decisions, in eight states by state legislature and in three states by popular vote. Clearly, the courts played an important role in the process of legalization of same-sex marriage. The opinion of the senators on this issue has also shifted to support over the years. The first polling vote among the senators was on 10 September 1996, where the topic was to introduce Defense of Marriage Act (DOMA), which defines marriage as the union between one man and one woman and to prohibit same-sex marriage. A total of 14 senators among 100 were against DOMA; however, only four supported same-sex marriage. Since then, the opinion of the senators on this issue has evolved, and in 2014 there were 57 senators who were in support of same-sex marriage. State legislatures, popular vote and the courts have made remarkable changes over the past two decades in laws defining whether marriage is limited to relationships between a man and a woman or may be extended to same-sex couples.

The public support for gay rights has also increased over the years [2–4]. A Gallup poll demonstrates this shift [5]. For instance in 1996, 68% of individuals in the United States opted to vote against the legalization of same-sex marriage, whereas in 2016, 61% of individuals opted to vote in favour of legalization of same-sex marriage. Religious affiliations have substantially influenced the increasing public support for same-sex couples, as demonstrated in a study of data until 2010 [2]. However, political affiliation has stronger influence (almost five times more) as compared to the religious affiliations [2]. The major shift in opinions among individuals has been attributed to increased interaction with same-sex couples via interpersonal [6–9] or parasocial contacts [10,11]. Recent support is also due to younger people favouring legalization of same-sex marriage compared to older generations [12].

Although significant studies have been done to determine the factors that play important roles in the increasing public support, research on the evolution of senators’ opinions, state-law on the issue of same-sex marriage and the relationships between these two indicators of public opinion is limited. A first study on the diffusive process underlying senators shifting from opposing to supporting same-sex marriage can be found in [13], but only a limited dataset is considered and the roles of the individual factors mitigating the diffusion of opinions are not isolated. Specifically, this study calculates a likelihood for senators in 2013 to change their opinions based on personal characteristics (e.g. political party, re-election status), home state voting history and laws (e.g. previous presidential election results, state-wide legalization of same-sex marriage), interactions with other senators (e.g. the number of senators who announce support for same-sex marriage that year) and political climate (e.g. announcements by the president and US Supreme Court) [13]. Here, we explore the opinion formation of senators and state-law as they evolve over 19 years preceding the national legalization of same-sex marriage and attempt to identify factors mitigating interactions between senators and states that may have affected the dynamics. In other words, we neglect individual variations among senators and states and seek to define interaction patterns that best describe the observed opinion dynamics.

Interactions between agents drive opinion dynamics in real-world scenarios, which have been studied from both modelling and data-based perspectives. In the literature, a variety of mathematical models have been proposed to study opinion formation in a social group. These mathematical models incorporate protocols motivated from real-world phenomena and are important to understand the dynamics of opinion formation and for their predictive power. A review of such mathematical models can be found in [14]. For example, the ‘voter model’ [15–18] represents the voters and their interactions as nodes and edges, respectively, in a network and the conditions to reach consensus are studied. Other well-studied models include the ‘majority rule model’ [19–21], where the agents share binary opinions and group decision is determined by the position of the majority; the ‘compromise model’ [22], where the agents interact randomly in a pairwise manner, and update if their opinions are within a given threshold; the ‘Deffuant model’ [23], where the agents share continuous, rather than discrete binary, opinions and interactions occur if two agents’ opinions are sufficiently close to each other; the ‘Sznajd model’ [24], which assumes that the opinion of an agent is driven by the opinions of its neighbours; and the ‘social impact model’ [25,26], which is based on a psychological theory that considers the influence of the group size, their convincing power and the distance from a target opinion. Here, distance can refer to either physical (spatial proximity) or psychological metrics (closeness in terms of personal opinion).

In addition to the theoretical exploration of these mathematical models, the recent availability of large datasets has motivated the empirical analysis of opinion dynamics based on the real-world phenomenon, for example, Indian [27] and Brazilian elections [28]. To study the Brazilian election results, a network-based model is applied along with the assumption of the Sznajd model, in the sense that a small group of individuals influences the opinion of their nearest neighbours if and only if they share common opinions among themselves. The model shows a good agreement with the observed voter distribution from the Brazilian elections dataset [29]. Furthermore, a prospective experiment has been performed to demonstrate that opinion formation among individuals is subject to social influences [30]; for example, when an individual lacks knowledge, the more susceptible she is to seek information from others to update her beliefs. Similarly, in an online experiment [31], three main types of individuals are identified based on opinion update processes, namely stubborn individuals (who do not change their opinions), compromising individuals (who negotiate with their neighbours’ opinions) and conforming individuals (who ignore their own opinion and take on opinions of their neighbours). For group-level behaviour, a study of synchronized clapping in a concert hall uses a ‘random field Ising model’ to find that the emergence of collective behaviour is induced by a combination of imitation and social pressure [32]. Opinion formation using different kinetic models is further studied in [33], where using the presidential election results in the state of Arizona, it has been shown that the citizens tend to live in a neighbourhood with similar political belief. The above-mentioned real-world examples suggest that opinion formation at the individual and group level is the result of a complex negotiation based on multiple social factors. Public policies are perfect examples of such complex opinion formation that aggregates interactions across multiple social scales. Consequently, we target the timely issue of same-sex marriage in the United States to provide evidence that the geographical and ideological similarities are significant contributors to senators’ opinions and state-law adoption.

In the present work, we study the issue of same-sex marriage from data on state-law adoption and senatorial support from 1996 to 2014. We perform a correlational analysis to capture interdependence between these data and factors that may mitigate opinion dynamics. We hypothesize that two external factors impact the dynamics of the state-law and the senators’ opinions, namely topological distances between the states, which capture the influence of geographical proximity, and state-government ideology, which condenses beliefs driving social and political policy. We investigate the effect of these factors on both the state-law and the senators’ opinions using distance-based correlations. Distance-based correlation, as defined in Gallos *et al.* [34], has been an effective tool to unfold the topological dependencies of real-world phenomena, for example, the spread of obesity [34], cascading failures [35], motor vehicle deaths [36] and traffic percolation [37]. Defining distance in terms of topology and ideology, we anticipate that both the state-law, and the senators from states that are topologically closer exhibit comparable trends. On the other hand, we expect that the senators sharing similar ideology display comparable patterns, while sorting states by ideology may provide weaker correlation among their laws. Drawing on the results of the data-based analysis, we model the evolution of state-law using a distance-based social impact model, with which we seek to identify when states switch from banning to legalizing same-sex marriage. The present study seeks to uncover and model this real-world phenomenon and has two-fold contributions. First, we unravel the components that may have affected the opinion dynamics, and second, we build a protocol which captures these driving factors and verify its predictive power with the real-world dataset. The two-way approach of the problem not only identifies the presence of driving factors, but also validates the dynamics in a predictive model.

## 2. Results and discussion

### 2.1. State-law and senators’ opinions

State-law and senators’ opinions reflect the position of the individual states and the senators, respectively, on the issue of same-sex marriage which we categorize as ‘support’, ‘oppose’ or ‘ambiguous’ for the senators and ‘legal’, ‘ban’ or ‘neither legal nor ban’ for the state-law. The definition of these terms and the methods for data collection are further elaborated in the Methods section. We assign values for the state-law of state *i* for year *t* as *x*_*state*_(*i*,*t*)∈{−1,0,1} if state *i* has legalized (*x*_*state*_=1), banned (*x*_*state*_=−1) or neither legalized nor banned (*x*_*state*_=0) same-sex marriage. Similarly, we assign values to the senators’ opinions for each year from 1996 to 2014 as 1,−1 or 0 if the particular senator supports, opposes or is ambiguous on the issue of same-sex marriage. Averaging the opinions of the two senators representing each state where each senator can assume values from {−1,0,1}, we define *x*_*sen*_(*i*,*t*)∈{−1,−0.5,0,0.5,1} as the average senators’ opinions for state *i* in year *t*.

**Figure 1.**
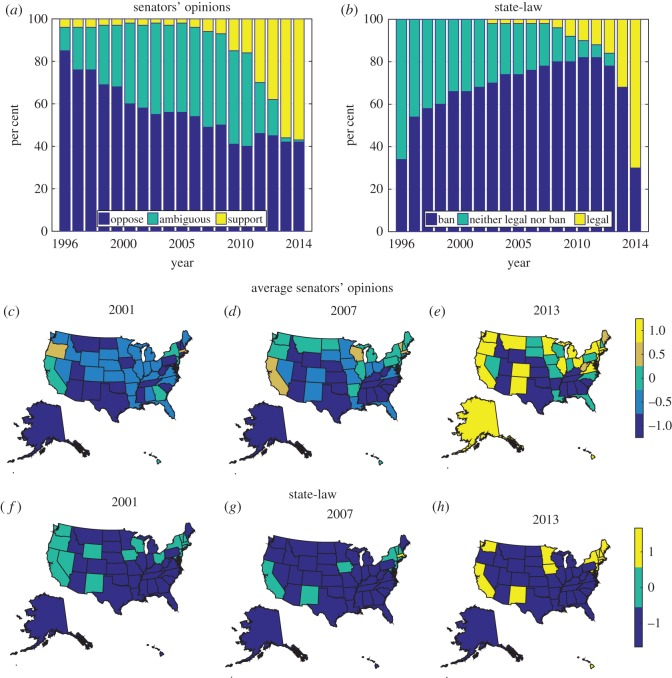
Distribution of (*a*) senators’ opinions and (*b*) state-law on the issue of same-sex marriage over time. (*c*–*h*) Snapshots of the data for three representative years, 2001, 2007 and 2013, showing (*c*–*e*) average senators’ opinions and (*f*–*h*) state-law. Both the bar plots and the maps are created using Matlab, version R2016a (https://www.mathworks.com/).

The data are shown in figure 1*a*,*b*, where we present the distribution of state-law and senators’ opinions over time, and in figure 1*c*–*h*, where we present snapshots of the data for three representative years, namely 2001, 2007 and 2013. We observe in figure 1*a* that ambiguity among the senators first grows from opposing same-sex marriage and then converts into support in recent years. However, figure 1*b* shows that strong laws emerge monotonically over time either in support of or banning same-sex marriage. Comparing figure 1*a*,*b*, we see that support among the senators initiated earlier than the state-laws to admit same-sex marriage.

### 2.2. Distance-based correlation

**Figure 2.**
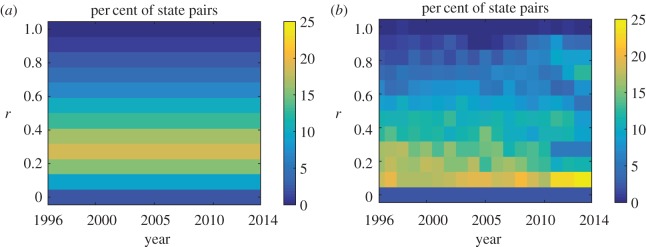
Percentage of state pairs that are separated at a given distance, *r*, computed based on (*a*) topological distance and (*b*) ideological distance from the years 1996–2014.

Following from Gallos *et al.* [34], we compute a distance-based correlation function to quantify the influences of two distance metrics on the senators’ opinions and the state-law. The two distance metrics are defined in terms of the geographic topology and state-government ideology. For the topological distance, the states are considered as nodes in a network and pairs of states sharing physical boundaries are linked by an edge. Then, the topological distance between a pair of states is defined as the shortest path between them over this network. On the basis of the geography of the United States, the maximum distance obtained between a pair of states is found to be 11, which we use to normalize all topological distances *r* between 0 and 1. The percentage of the state pairs that are separated at a topological distance *r* is presented in figure 2*a*.

The ideological distance is defined as a metric in [0,100] based on state-government ideology from the database in [38], which is further described in the Methods section. The normalized ideological distances are combined in 12 equally spaced bins between 0 and 1 to allow comparison with topological distances and are presented in figure 2*b*. Since the topological distance is fixed, the percentage of state pairs separated at a given distance is constant over time, whereas the ideology being dynamic has percentages that change over time. Selecting either topology or ideology to set the normalized distance *r*∈[0,1], we define the distance-based correlation function *C*(*r*) introduced in [34] as

**Figure 3.**
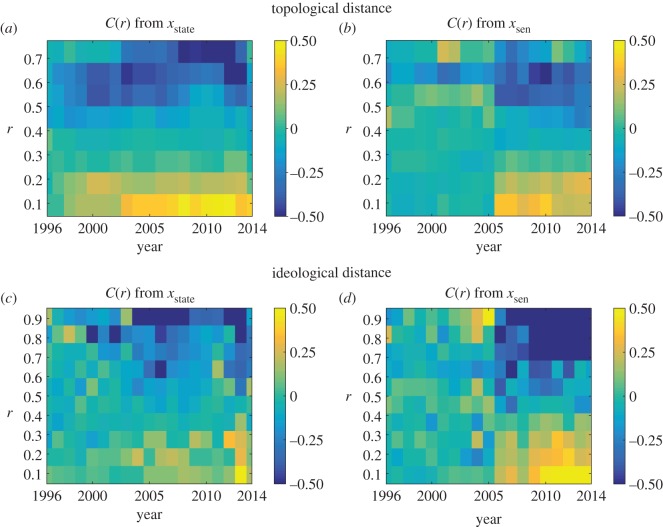
Distance-based correlation for (*a*) state-law and (*b*) average senators’ opinions computed using topological distances with *r* varying from 0 to 0.7. Distance-based correlation for (*c*) state-law and (*d*) average senators’ opinions computed using ideological distances with *r* varying from 0 to 0.9.

2.1 $$C(r,t)=\frac{1}{\sigma {\left(t\right)}^{2}}\frac{\sum_{i,j=1}^{N}\left(x_{*}(i,t)-{\bar{x}}_{*}(t))(x_{*}(j,t)-{\bar{x}}_{*}(t))\delta (r_{ij},r\right)}{\sum_{i,j=1}^{N}\delta (r_{ij},r)},$$

where *N* is the number of states used in the analysis, x_*_ is either *x*_*state*_ or *x*_*sen*_, ${\bar{x}}_{*}$ and *σ*^2^ are the mean and variance of x_*_ over all states in a given year, respectively, *r*_*ij*_ is the measured distance between state *i* and state *j* in terms of either ideology or topology and the function *δ* is the Kronecker delta that selects states at distance *r* as set by the argument of the function *C*. It is worth mentioning that *r*_*ij*_ is constant for all years when topological distances are considered; however, its value changes in the case of ideological distances. Similar to the commonly used Pearson correlation coefficient, large values of *C*(*r*) denote strong correlation among states separated by a distance of *r*, and values close to zero denote weak correlation among these states. Also, positive values of *C*(*r*) imply positive correlations, while negative values indicate negative correlations. Figure 3 shows the distance-based correlation coefficients for state-law and average senators’ opinions computed using topological and ideological distances.

*When distance is defined in terms of geography, pairs of states and senators separated by short distances show positive correlations, with more pronounced correlations for state-laws than senators’ opinions*. In figure 3*a*,*b*, we observe that both the state-law and the average senators’ opinions display positive correlations at *r*=0.1 and *r*=0.2. Moreover, the small-distance positive correlations generally increase in time, initiating earlier in the state-law than the average senators’ opinions. In addition, state-laws show stronger correlations than senators as time increases. Hence, nearby state pairs are more similar both with respect to state-law and average senators’ opinions in recent years. However, correlations decrease as the topological distance increases in both cases and goes to zero at about *r*=0.4 for the state-law, and at *r*=0.3 for the average senators’ opinions. At *r*>0.5, we observe long-distance negative correlations in both cases; however, these are more pronounced in the state-law in recent years. We note that *r* is varied from 0.1 to 0.7 since the number of state pairs which are topologically distant from each other at *r*>0.7 is very small (see figure 2*a*). Specifically, less than 8.16% of state pairs are separated by distances greater than *r*=0.7. These results suggest that the impact of the physically proximal states is evident in both state-law and senator’s opinions, but is more pronounced in state-law.

*When distance is defined in terms of ideology, pairs of states and senators separated by short distances show no positive correlations except for senators in recent years; long-distance pairs show negative correlations in both cases*. Figure 3*c* shows that the state-law for the states with similar ideology displays correlation near zero. However, small-distance positive correlation is strong for average senators’ opinions in the year range 2007–2014 as seen in figure 3*d*. In the same year range, we also observe long-distance negative correlation among the senators who are ideologically far from each other. It is noteworthy that this negative correlation should not be neglected since the number of state-pairs that are ideologically distant are not trivial as seen in figure 2*b* (20.88% of state pairs averaged over years 2008–2014 are separated by distances greater than *r*=0.7). These results indicate that state-government ideology has a significant influence on the opinion of the senators in recent years. In particular, the senators who are ideologically close hold similar opinions on same-sex marriage, whereas those who are ideologically distant have different viewpoints. This long-distance negative correlation has also been captured to some extent in the state-law and starting earlier (starting from year 2000) than that of the average senators’ opinion.

To summarize, we observe recent polarization in the state-law when topological distances are used (figure 3*a*), and in the average senators’ opinions when ideological distances are used (figure 3*d*). Additionally comparing figure 3*a*,*d*, polarization is more extreme in the average senators’ opinions with ideological distances.

### 2.3. Control experiment

To further verify the results in terms of the correct identification of the factors, we randomly permute the values for the time series of both the state-law and the senators’ opinions within each year. This ensures that the mean and standard deviations used in equation (2.1) are maintained, but the individual state and senator dynamics are scrambled. We compute the distance-based correlation *C*(*r*,*t*) of the scrambled data considering both topological and ideological distances.

**Figure 4.**
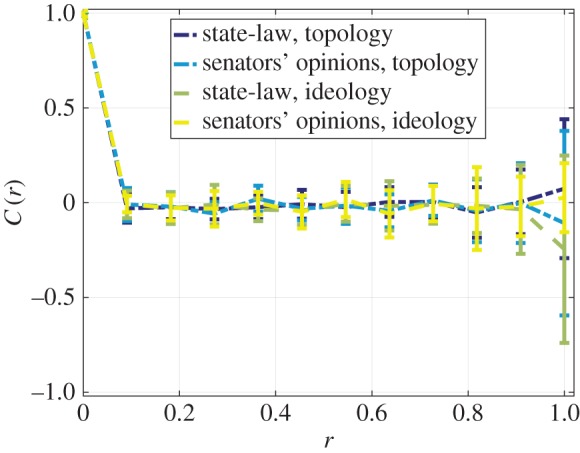
Mean distance-based correlation values for randomly permuted state-law and senators’ opinions with respect to the states using both topological and ideological distances. Points indicate the mean value, and error bars denote one standard deviation over the entire year range.

*The observed effects for state-laws and senators’ opinions result from interactions, rather than an average change in support or legalization*. The results as shown in figure 4 demonstrate that the distance-based correlation converges to zero immediately from *r*=0.1 for both state-laws and senators’ opinions using ideological and topological distances. This verifies that *C*(*r*,*t*) captures individual and interactional dynamics beyond a mere increase in overall percentage support for same-sex marriage. This suggests that topological and ideological factors provide an explanation for the previously observed correlations.

### 2.4. Mutual dependence of the state-law and the average senators’ opinions

To study the interdependence of the state-law and the average senators’ opinions, we compute the cross-correlation of the two time series after lagging them in time. Other existing tools that can be used to detect the interdependence include transfer entropy [39–43] and event synchronization [44]. However, we choose cross-correlation as it has been used to detect the directionality of opinion flow in a real-world phenomenon [45]. For this analysis, we concatenate the time series of either state-law or the average senators’ opinions from each state to build an amalgamated dataset. For example, to build the one-year lagged time series of the state-law from 1996 to 2013, we first delete the values of all the 50 states of the year 2014, then we concatenate the time series of each state in succession, starting from Alabama ending with Wyoming. It is important to note that here we include Alaska and Hawaii, as the cross-correlation analysis is independent of whether or not states share boundaries with each other. In a similar fashion, we build the dataset for the average senators’ opinions. To compare one-year lagged state-law with unlagged average senators’ opinions, we compute the Pearson correlation coefficient *R* of the state-law from 1996 to 2013 with that of the average senators’ opinions from 1997 to 2014 for all the 50 states. This process is repeated for lags of 5 years on both the states and senators.

**Figure 5.**
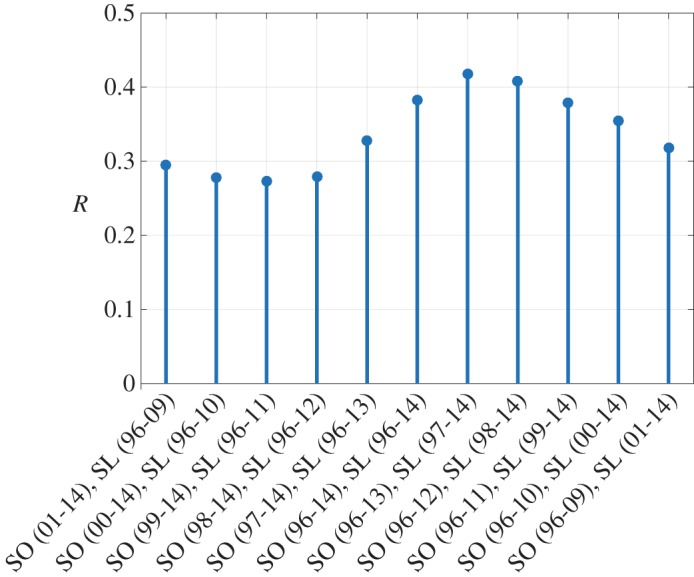
Correlation coefficient of the lagged time series of the average senators’ opinions and the state-law. The horizontal axis denotes the two time series that are compared; ‘SO’ stands for average senators’ opinions, ‘SL’ stands for state-law and the time ranges of the two times series are in parentheses.

**Figure 6.**
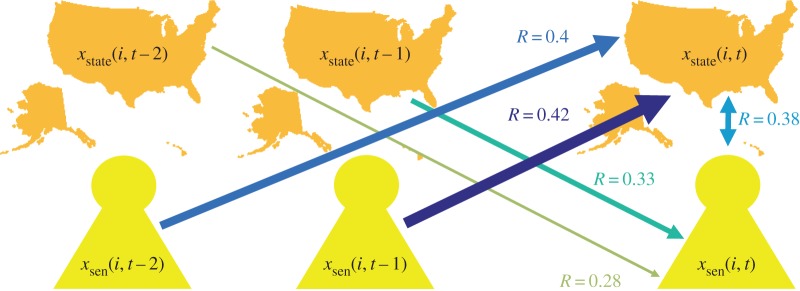
Schematic representation demonstrating the correlation coefficients for state-laws and senators’ opinions with lags of one year, two years and without any lag. The correlation coefficient is maximum when one-year lagged average senators’ opinions (from 1996 to 2013) are compared to state-law (from 1997 to 2014), with a value of *R*=0.42. Maps are created using Matlab, version R2016a (https://www.mathworks.com/).

*State-laws show maximum correlation with senators’ opinions lagged in time, suggesting that opinions flow from senators’ opinions to state-laws*. Figure 5 presents the correlation coefficient for the two time series, when compared with different year ranges. Interestingly, the correlation coefficient is maximum when state-law time series, chosen from 1997 to 2014, is compared with the average senators’ opinions time series, chosen from 1996 to 2013. In other words, the state-law time series shows maximum dependence on the time series of the average senators’ opinions with a difference of a year when the state-law is allowed to follow the senators’ opinions. A summary of results is shown in figure 6, which shows the correlation values for lags of one year, two years and without any lag. For the unlagged version, a two-way directed arrow is used to represent the mutual dependencies on each other, whereas for the lagged version a one-way directed arrow is used to show the influence of the past values of either state-law or average senators’ opinions on that of the present values.

### 2.5. Social impact model

From the dataset, we observe that the state-law and the average senators’ opinions are both dependent on the topological distances, average senators’ opinions are dependent on the ideological distances and the state-law shows maximum dependence on the average senators’ opinions from one year prior. Hence, we seek to model the state-law as it varies with both the topological distances and the average senators’ opinions. We select a distance-based opinion dynamics model drawing on impact theory [26] to study the dynamics of the state-law in terms of initiation of legislation legalizing same-sex marriage. With this motive, we classify the state-law in two groups as ‘legalized’ and ‘not-legalized’ same-sex marriage, thus eliminating the possibility of ambiguity used in the previous analyses.

In the model, state *i* in year *t* assumes binary opinions ${\hat{x}}_{\mathrm{state}}(i,t)=\{-1,1\}$, which correspond to states where same-sex marriage is legalized (${\hat{x}}_{\mathrm{state}}=1$) or not legalized (${\hat{x}}_{\mathrm{state}}=-1$). To capture the social pressure that state *i* experiences from all other states in year *t*, we define the impact *I*(*i*,*t*) given by

2.2 $$I(i,t)=\left[\sum_{j=1}^{N}\frac{1}{{r_{ij}}^{\alpha }}(1-{\hat{x}}_{\mathrm{state}}(i,t){\hat{x}}_{\mathrm{state}}(j,t))\right]-\left[\sum_{j=1}^{N}\frac{1}{{r_{ij}}^{\alpha }}(1+{\hat{x}}_{\mathrm{state}}(i,t){\hat{x}}_{\mathrm{state}}(j,t))\right],$$

where *N*=48 is the number of states used in the model (omitting Hawaii and Alaska because they do not share physical borders with any other states), and *α* is a constant exponent that indicates how fast the impact decreases with the pairwise state distances *r*_*ij*_. In our model, we choose *r*_*ij*_ as the topological distances that have been used in the distance-based correlation analysis, which has been shown to influence state-law. Note that the first sum in equation (2.2) denotes a persuasive impact from the states who hold opposite opinions than state *i*, and the second sum denotes a supportive impact from the states who share the same opinions. By definition, positive impact *I*(*i*,*t*)>0 signifies a pressure in favour of opinion change for state *i*. The opinion then updates as follows:

2.3 $${\hat{x}}_{\mathrm{state}}(i,t+1)=-\text{sign}[{\hat{x}}_{\mathrm{state}}(i,t)I(i,t)+wx_{\mathrm{sen}}(i,t)].$$

In the traditional social impact model, the second summand in the argument of the sign function is reserved for a random variable representing noise. However, we use the average senators’ opinions here based on the relationship between senators and states evidenced by the time-lagged correlations.

We use this model to capture the dynamics of the state-law by identifying the states that initiate legalization of same-sex marriage with the two factors (topological distance and a year-lag average senators’ opinions) provided as the input parameters. To make the dataset consistent with the model parameters for comparison of results, we replace the zero values of *x*_*state*_ with negative ones in order to convert the data to binary values. We choose the initial condition for ${\hat{x}}_{\mathrm{state}}$ as the state-law for *N* states for the year 1996 and allow it to update for the next year following equations (2.2) and (2.3). We simulate the model for 18 years, that is, until the end of year 2014. The free parameters, *α* and weight *w*, are tuned to fit the model to the dataset by allowing *α* to vary from 0 to 10 with an increment of 0.05, and *w* to vary from −20 to 0 with an increment of 0.05. We compare the ${\hat{x}}_{\mathrm{state}}$ values with the binary state-law data for all states and for all years using root mean square error to quantify the error. The optimal ranges of the parameter values correspond to values that minimize the error.

**Figure 7.**
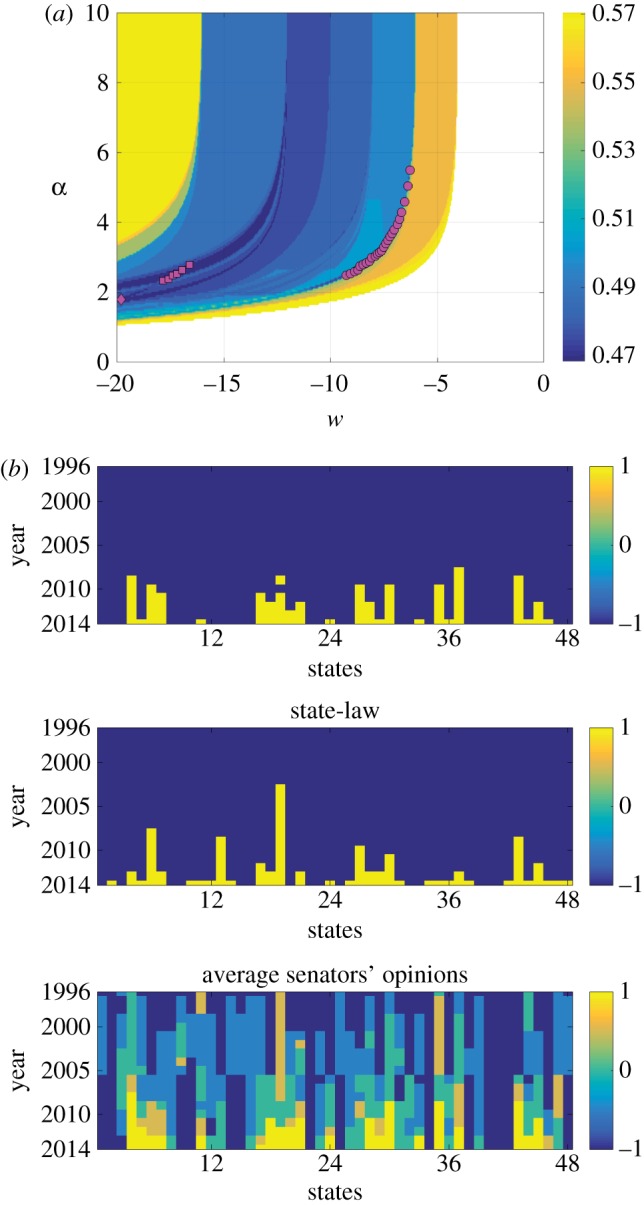
(*a*) Contour plot of root-mean-square error between model generated and state-law data, with weight *w* and *α* varying. Diamond (type I), squares (type II) and circles (type III) correspond to the minimum of the root-mean-square error equal to 0.468. (*b*) For comparison, ${\hat{x}}_{\mathrm{state}}$ of type II, state-law and the average senators’ opinions are plotted for all years and states.

To study the dependence of the root mean square error on *w* and *α*, we compute the contour plot in figure 7*a*. The root mean square error value equal to 0.585 corresponds to the case when the ${\hat{x}}_{\mathrm{state}}$ values are all equal to −1 for all years and for all states, which is considered as a control condition. In other words, the control condition is that ${\hat{x}}_{\mathrm{state}}$ values remain the same as the initial condition and never change. This condition sustains at positive values of *w*, whereas only the negative values allow ${\hat{x}}_{\mathrm{state}}$ to switch in equation (2.3), and initiate the legalization. To understand parameter values that give systems with less error than this control condition, the value 0.585 is set as a ceiling for the root mean square error. Figure 7*a* demonstrates the domain of the parameter values that result in improved error, where the root mean square error values equal to 0.585 are omitted and are set to white on the plot. The minimum value of the root mean square error obtained is equal to 0.468. Corresponding to this minimum value, we get three configurations of ${\hat{x}}_{\mathrm{state}}$ denoted by type I, type II and type III as shown in figure 7*a* with diamond, squares and circles, respectively. In figure 7*b*, we plot the ${\hat{x}}_{\mathrm{state}}$ values for type II configuration with *α*=2.35, and *w*=−17.8, and compare it with the state-law and the average senators’ opinions.

In figure 7*b*, we observe that the update protocol, (2.2) and (2.3), identifies states that shift from banning to legalizing same-sex marriage in the dataset. The model identifies 23 states that switch to legalizing same-sex marriage at the end of 19 years in comparison to 33 states with respect to the state-law data at the end of year 2014. However, there are two false positives, namely in states 20 (Michigan), and 33 (Ohio). This means that there are 12 states that the update protocol fails to identify, but of these, 11 states have switched from banning to legalizing same-sex marriage in 2014. We comment that the failure in identification of the 12 states may be because the protocol in a few states identifies the switch with an inherent lag. Overall, this simple model is capable of capturing the complex dynamics of the state-law adoption based on two input parameters, namely the topological distance and a year-lag average senators’ opinions, which we have determined to be the driving factors from the correlation analysis. Nevertheless, the physical significance of the optimal *α* and *w* values is as yet unclear.

To quantify the performance of the model by comparing all three optimal configurations over all years, we use sensitivity and specificity measures [46]. Sensitivity is defined as the ratio of the number of states legalizing same-sex marriage that the model correctly identifies (true positives) and the total number of states that legalize same-sex marriage from the dataset in a specific year. On the other hand, specificity is defined as the ratio of the number of states not legalizing same-sex marriage that the model correctly identifies (true negatives) and the total number of states that do not legalize same-sex marriage from the dataset in a specific year. Sensitivity and specificity measure the true positive rate and true negative rate, respectively, scaled between zero to one, where the value one corresponds to a perfect predictor model. The results are summarized in table 1. Note that the first time a state legalized same-sex marriage was in 2003, hence we do not have sensitivity values in prior years. Comparing the sensitivity values corresponding to each year, we see that types I and II perform similarly for all years, except in 2009, where sensitivity for type II is 0.5 in comparison to a value zero for type I. The sensitivity values for types I and II increase over years and are greater than 0.8 in the year range 2011–2013 denoting a reasonably good performance in identifying the true positives. However, for type III, sensitivity values are small in 2010 and 2011 and gradually increase over years. For each type, we observe that the specificity values are greater than 0.8, in particular type III has values close to one for all years. In other words, type III is almost perfect in identifying the true negatives. Note that, in each type, sensitivity values decrease in 2014, since there were 18 states that actually legalized same-sex marriage in comparison to a total of 15 states in the end of 2013. Synthesizing these results, we can see that, while opinion formation is more complex in reality and certainly has random components, this simple update protocol works well in predicting the states that switch from banning to legalizing same-sex marriage.

**Table 1.** Sensitivity and specificity values for type I, II and III configurations from 1996 to 2014.

year	1996–2002	2003–2007	2008	2009	2010	2011	2012	2013	2014
sensitivity (type I)	n.a.	0	0	0	0.60	0.83	0.88	0.87	0.64
specificity (type I)	1	1	0.98	0.96	0.91	0.9	0.85	0.94	0.87
sensitivity (type II)	n.a.	0	0	0.50	0.60	0.83	0.88	0.87	0.64
specificity (type II)	1	1	0.98	0.96	0.91	0.88	0.83	0.94	0.87
sensitivity (type III)	n.a.	0	0	0	0.20	0.33	0.50	0.67	0.40
specificity (type III)	1	1	1	1	1	1	0.98	0.97	1

We directly compare the simulation results and with the data in figure 7*b*, where we also add the average senators’ opinions. These results show that the inclusion of the average senators’ opinions in our update protocol does not solely govern the ${\hat{x}}_{\mathrm{state}}$ values. We observe noticeable discrepancies between the ${\hat{x}}_{\mathrm{state}}$ values and the average senators’ opinions, confirming that the result is driven by both the distance-based measure of the state-law and the average senators’ opinions.

## 3. Conclusion

The present study supports the hypothesis that physical proximity has a strong influence on both the state-law and the senators’ opinions, whereby the states and the senators that are close in terms of geography tend to have similar state-law and senators opinions, respectively, whereas the states that are far tend to have different state-laws. In addition to the physical proximity, the state-government ideology is also found to be a predictor of the senators’ opinions, since the senators who share close ideology had similar opinions and the senators who are ideologically distant had opposite opinions. The effect of state-government ideology on the state-law is observed in terms of long-distance negative correlation, specifically that the state-law in the states that are ideologically far have different state-laws. However, states with similar ideologies do not have similar laws. All these results are more pronounced in recent years. In addition, the analysis reveals that the state-law shows maximum dependence on the senators’ opinions from one year prior. A distance-based social impact model is able to capture the evolution of the state-laws and shows a good predictive power in terms of correctly identifying the states that switch from banning to legalizing same-sex marriage. To summarize, we understand public policy adoption is generally complex and depends on a variety of external factors. In our present study, we target same-sex marriage to identify these factors, which in future may be considered to understand or model other public policies that propagate via social and political change.

## 4. Methods

Here, we explain in detail how we build the dataset for the senators’ opinions and the state-law. There are two senators representing each state and, when combined for 50 states, there are 100 senators nationally. The senators may be reassigned at least every four years through the election process. For each year from 1996 to 2014, we assign values 1, −1 or 0 to each of these senators if the particular senator supports, opposes or is ambiguous, respectively, on the issue of same-sex marriage in that year. A particular senator is said to be ambiguous if he/she neither supports nor opposes same-sex marriage. We only consider the senators during their period of tenure, and after that we consider the opinion of the new senator, modifying the opinion value accordingly. We choose 26 December of each year to log the opinion of the senators for that specific year, regardless of whether a senator is reassigned within the year. The data are collected from the list of supporters of same-sex marriage from different sources (for example, personal candidate/senator websites, news media and Wikipedia) and each supporter is identified with the first mention of support, opposition or ambiguity publicly on this issue. If a particular senator is identified as a supporter, then, for all successive years, the senator is considered as a supporter until the end of his or her tenure. We rely on the available data over the Internet on news and senators’ websites to build the dataset.

The data for the state-law on the issue of same-sex marriage across 50 states are collected from Internet sources. Similar to senators’ opinions we assign values for state-law as 1,−1 or 0 if that particular state has legalized, banned or neither banned nor legalized same-sex marriage, respectively.

We define distances between states based on two measures: a topological measure, which is based on the geography of state borders, and an ideological measure, based on the political ideology of state officials. The topological data used in the present study is based on building a network where states are vertices and the states sharing a common physical boundary are linked with an edge. Shortest paths over this network define topological distances between state pairs. We exclude Alaska and Hawaii from the distance-based correlation analysis using topological distance since neither share physical boundaries with the remaining states.

The state-government ideology data that we use in our present study are created by Berry *et al.* [47]. The data are updated through 2013 and can be found in [38]. We use the ADA/COPE measure of state-government ideology for our study. For the year 2014, we use the state-government ideology same as that of 2013. The state-government ideology data measure the mean position of the elected public officials in a state on a liberal–conservative continuum, weighted based on the power they have over public policy decisions. The score ranges from zero, representing the most conservative value, to 100, representing the most liberal position. Similar to topological distances, we normalize the score between zero and one for the ideological distances. Note that we exclude Alaska and Hawaii in our study where we use topological distance, particularly in the distance-based correlation analysis and in the model, because it is defined based on whether states share physical boundaries or not. However, we retain these two states in cross-correlation analysis and in the distance-based correlation analysis using ideological distance, which is independent of this fact. The numerical analysis is performed using Matlab, version R2016a.

## Data accessibility

The data for the senators’ opinions and the state-law are deposited at and available from VTechData [48].
